# Snake Poison from a Chemico-Physiological Point of View

**Published:** 1887-01

**Authors:** T. Wesley Mills

**Affiliations:** M. A., M. D., L. R. C. P., London. Professor of Physiology, McGill University, Montreal


### ART. IV.—SNAKE POISON FROM A CHEMICO-PHYSIOLOGICAL POINT OF VIEW.

BY T. WESLEY MILLS, M. A., M. D., L. R. C. P., *London.*

*Professor of Physiology, McGill University, Montreal.*

It has occurred to me that an account of the careful and thorough researches made recently into the nature of snake-poison might prove interesting to the readers of this JOURNAL and form a fitting sequel to the graphic paper of G. A. Stockwell, which appeared in the October number.

That the real nature of serpent venom should so long have been overlooked was largely owing to the stereotyped nature of our conceptions as to the class of bodies to which a poison must, as was thought, belong.

That a poison might be neither an acid nor an alkaloid, if of an obscure nature, was scarcely dreamed of till very recently, even by chemists and physiologists. A Commission was appointed to investigate snake-poisoning so far as India and Australia were concerned, which reported in 1874. Their work was valuable and suggestive, but incomplete, especially from the chemical side.

The first valuable chemical investigation of serpent venom was made by Prince L. Bonaparte, in 1843. After examination of the virus of an adder (pelias verus), he concluded that the essential poison was associated with the coagulum produced by alcohol.

In 1861 Weir Mitchell obtained from rattlesnake poison an "albuminoid body," which he named Crotaline, by treating the filtrate from the boiled venom with alcohol.

It would appear then that Weir Mitchell was the first to recognize what the latest investigation have made perfectly clear—that snake venom is a proteid body. A chemical analysis of the venom by Armstrong, in 1873, proved it to consist of C. H. N. O. S. and P., which, so far as it went, was in favor of its being a proteid.

But the whole matter was in a most unsatisfactory state till quite recently, when it was very thoroughly investigated chemico-physiologically by Weir Mitchell and Reichert in America, and Wolfenden in England; and it is gratifying to find that upon essential points these independent investigations have arrived at the same conclusions. Weir Mitchell and Reichert have worked conjointly upon the virus of the American rattlesnake, the moccasin and the copperhead; Wolfenden on that of the Indian cobra and the Indian viper.

From the rattlesnake and the moccasin the first two observers obtained three proteids; of these they consider one to be a *peptone*, another from moccasin venom they rank as a *globulin.*

From this venom Wolfenden obtained a globulin, but only traces of peptone.

He, however, disputes the grounds on which the first named investigators base their conclusions as to the nature of their "peptone" and "globulin," and would place the substances which they thus designate among bodies intermediate between true albumins and peptone.

Notwithstanding this difference as to details, the fact remains that these observers are agreed that the active principles of snake venom are proteid and as proteid in character.

As fresh snake venom is acid in reaction, investigators were led to look for some special acid which should account for its toxic properties.

Winter Blyth, in 1876, prepared and figured what he called cobric acid, and considered the sole active principle. Wolfenden recently undertook, by careful examination, to settle the matter. He has shown that there is no such acid; that the crystals of Blyth are really sulphate of lime, and suggests that the acid reaction of the venom may be due to the presence of *acid albumin*, at least in some cases.

The specimens of cobra poison examined contained fat; and crystals of fatty acid were obtained. Wolcott Gibbs had previously negatived Blyth's view in regard to "cobric acid.

But all three of these observers are agreed as to the absence of any alkaloid whatever. It has been further shown that the toxic action of snake venom is not due to the presence of any microbe, nor any ptomaine (if it be necessary to mention the latter after stating the absence of alkaloids).

If acids, alkaloids and germs may be excluded, of what then does the power consist?

Cobra poison consists solely of proteid matter and of the following kinds:

1. Globulin, always present; probably kills by interference with the respiratory mechanism (asphyxia)—without paralysis—causing local inflammation, but not of great intensity compared with viper venom.

2. An albumin resembling *acid albumin*, which is precipitated along with globulin by saturation with a neutral salt and which is in some degree dialysable. This body acts probably on the respiratory apparatus, chiefly like globulin, but less powerfully.

3. An albumin precipitated by sodium sulphate from the magnesic filtrate, and which is *serum albumin*. This produces ascending paralysis, ending fatally by interference with the muscles of respiration.

4. Traces of *hemialbumose* and questionable traces of peptone. "I regard these as accidental, and probably due to the length of time the poison has been kept" (Wolfenden). In viper poison was found also the preponderating globulin, serum albumin and a body allied to acid albumin, probably an albumose. Weir Mitchell and Reichert found in their venoms globulin, albumoses and peptone, though the presence of this latter body is not beyond dispute.

These conclusions have been arrived at by the use of methods which, so far as can be seen at present, are almost perfect; and these researches, viewed historically, demonstrate the importance of thoroughly reliable methods. Details would be out of place in such a paper as this; but suffice it to say that the methods are not wholly new, but have been tried in other fields of investigation.

It is now clear why other investigators, using methods less reliable, came to the conclusion that the toxic action of snake venom was due to some alkaloid, acid, etc.

The fact was that these observers had in their filtrate bodies which were really proteid, but which at that time were not suspected of bearing that character, because the chemical properties of that class of bodies were then much less perfectly understood. These recent investigations show that it is most difficult to prevent some of these proteid matters from passing through filters and dialysers. They clearly demonstrate that the toxic action is due to the presence of a proteid in solution (not suspension); that in the absence of the proteid the effect does not follow; and that the effect is proportional to the quantity and kind of proteid present. All this has been actually shown by *experiment* and is not a priori inference.

In order to test the action of these bodies, injections were made into the blood of various animals and the results carefully noted.

The ordinary symptoms of the Indian viper poisoning are not constant but may cause death in one of three ways:

1. Primary convulsions.
2. Convulsions and paralysis.
3. Blood poisoning.

Its local effects are very marked, gangrene not being uncommon.

In viper poisoning there may be early cardiac embarrassment and speedy fall of blood pressure, sufficient even to cause death of itself.

It is well known that acid albumin is formed during peptic digestion; that globulin and serum albumin occur in blood serum and as Kühne first showed, the albumoses also are formed during artificial digestion, at least, while peptone has long been recognized as a final product of proteid digestion.

At first one is somewhat startled to find that these very bodies belong to the same class as those constituting the yellowish poison of serpents.

But peptoric, if absorbent at all as peptone from the alimentary canal into the blood, enters very slowly, and may possibly be changed into some other proteid. Our physiological chemistry has advanced far enough to show that there are globulin and globulins, etc.; that is to say bodies which while having much in common yet show chemical and still greater physiological differences. And, after all, our knowledge of the real chemical constitution of all proteid bodies is in a very crude state. Their molecular constitution is unknown. Many puzzling questions arise in connection with this and other topics it suggests.

What most impresses me is, on the one hand, the crudeness of our chemical knowledge and, on the other, the extremely delicate, we may say subtle, character of the mechanisms in a vital organism. But certain recent investigations of Pallitzer and others have brought us face to face with some new and impressive facts.

It has been shown that peptone, a peptone prepared by artificial digestion, and the albumoses prepared in a similar way, when injected into the blood of an animal produce *narcotic poisoning;* but curiously enough those bodies are without the slightest effect on some animals, as the rabbit. Pallitzer has shown that in several genera of animals, the *coagulability* of the blood is delayed or totally prevented; that the *blood pressure* sinks markedly, probably owing to voso-motor paralysis and manifesting itself chiefly in the splanchine area. The fact that the narcosis lasts only so long as the blood pressure is low, points to cerebral anæmia as its probable cause.

But peptone is never fatal while the kidneys are intact, for it is a diuretic and is eliminated rapidly; but a sufficiently large dose of the albumose is always fatal. Here then are examples of bodies, which, according to our chemistry, are very much alike, but which have a physiological action so different that one is fatal and the other not necessarily so. A body with identical ultimate chemical composition, even to the same quantity of each element may, as is well known, possess an entirely different molecular constitution with c[illegible]rrespondingly unlike properties and physiological action; hence it is possible to understand why the globulin of snake poison should have a different effect from that which we prepare in the laboratory.

It is, of course, open for us to believe that the toxic properties of snake venom may be due, not to the proteids it contains, but to something associated with these, but not yet isolated; for such a view there is at present no positive evidence. But assuredly there are more things in heaven and earth than are dreamt of in our philosophy.

It is true no antidote for snake poison has yet been found; but with the strides science is making, I am not hopeless that some way may be devised for the rescue of the victims of serpent bites, be it a genuine antidote or something else. The fact of the speedy and total ruin of hosts of blood corpuscles is serious; but if at a certain point this could be stayed and the poison neutralized or eliminated, the great creative power of the body, in the renewal of its tissues, including the red cells of the blood, might suffice to meet the emergency.

I should be inclined to hope and believe that man will ultimately prevail over the actual serpent as he is over the allegorical one.

